# Multi-spectral laser speckle contrast imaging for depth-resolved blood perfusion assessment

**DOI:** 10.1117/1.JBO.30.2.023517

**Published:** 2025-02-25

**Authors:** Liban Hussein, Sajjad Moazeni

**Affiliations:** University of Washington, Department of Electrical and Computer Engineering, Seattle, Washington, United States

**Keywords:** laser speckle contrast imaging, depth assessment, multispectral, multiple-wavelengths, skin burn characterization, non-invasive imaging

## Abstract

**Significance:**

Laser speckle contrast imaging (LSCI) is a widely used tool in biomedical imaging that leverages the interactions between coherent laser light and tissue to assess blood perfusion. Although effective for 2D imaging applications such as skin burn assessment and wound healing, conventional LSCI lacks depth-resolved capabilities, limiting its potential for deeper perfusion analysis. Enhancing LSCI for depth profiling would significantly expand its utility in applications such as vascular imaging and burn diagnostics.

**Aim:**

We investigate the use of multi-spectral laser speckle contrast imaging (MS-LSCI) for assessing blood perfusion at multiple depths, utilizing multiple laser wavelengths and advanced correlation techniques to improve depth localization.

**Approach:**

Two tissue phantom molds were fabricated to simulate blood vessels at varying depths. Laser wavelengths from blue to near-infrared (NIR) were used to perform controlled experiments. The visibility parameter, $V_{r}$, was employed to correlate and estimate the depth between the phantoms. In addition, a spectral wavelength mapping technique was implemented to enhance signal quality. Validation was conducted by imaging a human hand using the MS-LSCI setup.

**Results:**

MS-LSCI demonstrated improved depth profiling accuracy across varying laser wavelengths. The spectral wavelength mapping technique enhanced signal quality for wavelengths with limited penetration. The visibility parameter, $V_{r}$, provided consistent depth correlations across phantom models, with results validated through successful imaging of blood perfusion in a human hand.

**Conclusions:**

We highlight the potential of MS-LSCI for depth-resolved blood perfusion imaging using multi-wavelength approaches. The findings emphasize the technique’s feasibility for non-invasive biomedical applications, including burn wound assessment and vascular imaging.

## 1 Introduction

Non-invasive imaging techniques have revolutionized modern healthcare by offering valuable insights into tissue perfusion, blood/tissue oxygenation, and blood flow dynamics, which are critical for accurate diagnosis, treatment, and monitoring of various medical conditions.^1^^,^^2^ Among the non-invasive imaging modalities, laser speckle contrast imaging (LSCI) has emerged as a valuable tool. LSCI utilizes the speckle patterns that result from the interactions between laser light and tissue to extract information about blood flow dynamics and tissue perfusion. Through algorithms that examine the contrast and statistical properties of these speckle patterns, LSCI provides quantitative measurements of blood flow velocity and perfusion.^3^

LSCI offers several unique advantages over other non-invasive imaging techniques such as laser Doppler flowmetry (LDF) and optical coherence tomography (OCT). LDF measurements can only be taken from a single spatial location, leading to prohibitively long image acquisition times during beam scanning.^4^ Meanwhile, LSCI offers real-time imaging capabilities, allowing for faster and more continuous blood flow monitoring.^5^ OCT provides higher resolution cross-sectional imaging, but it has limited penetration depth,^6^ making it less suitable for assessing deeper tissue layers. LSCI, by contrast, has the potential to assess varying penetration depths. This is critical in applications such as burn depth detection, where it is used to distinguish between varying skin burn categories.^7^^–^^9^ Diffuse optical tomography (DOT) can also be used for non-invasive and depth-resolved blood oxygenation measurements;^10^^,^^11^ however, it cannot provide an accurate assessment of blood flow.^12^

In addition, LSCI imaging can be done with relatively simple commercial cameras and optical components, making this approach suitable for low-cost and small-form-factor biomedical devices.^13^^,^^14^ This simplicity reduces the overall cost of the imaging system and also allows for greater flexibility in the design and deployment of portable and handheld devices.

**Fig. 1:**
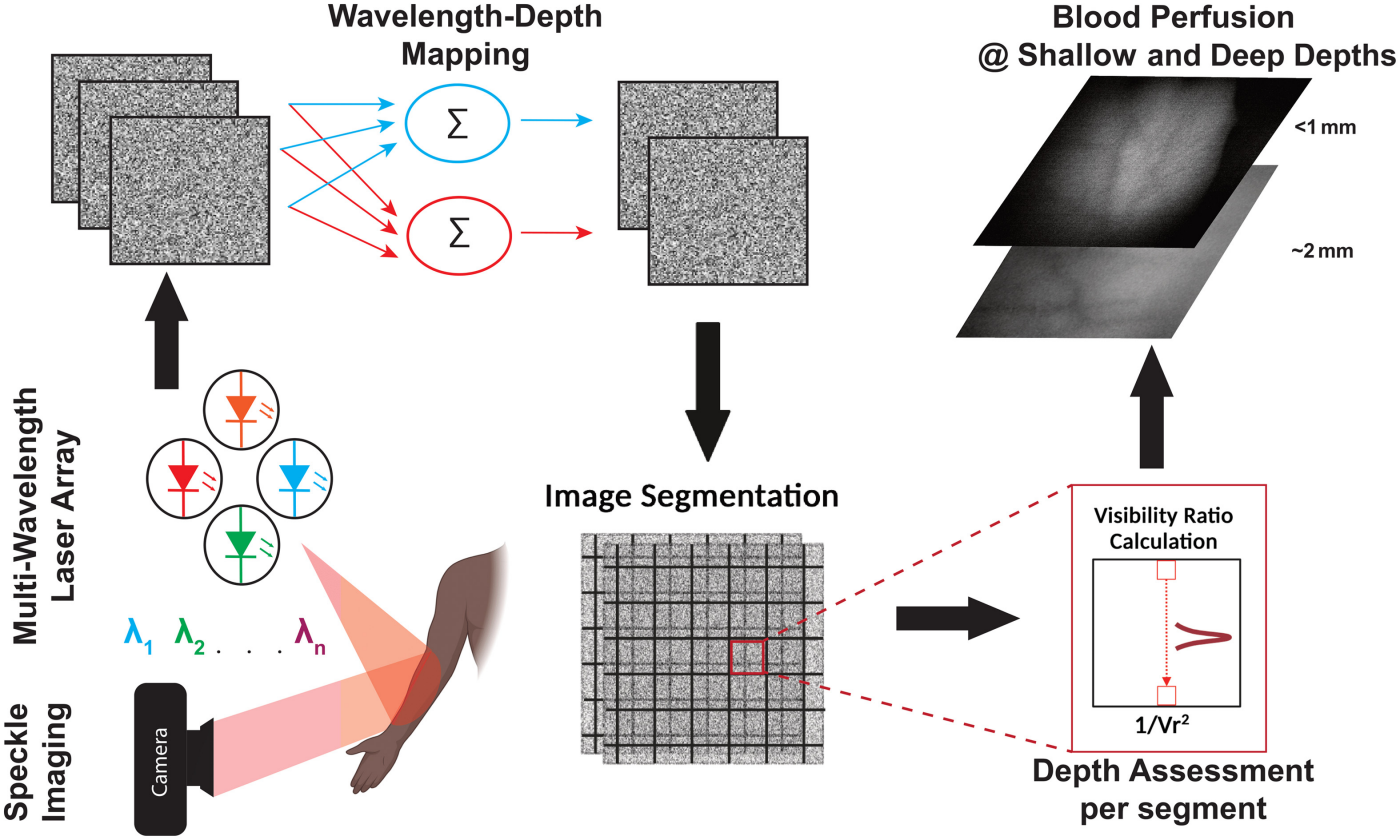
Overview of proposed framework for depth-resolved LSCI using multi-spectral LSCI (MS-LSCI).

Despite all the advantages of LSCI imaging, one major limitation of this approach is the lack of differentiating the depth of blood flow. This study presents a focused approach to enhancing depth assessment capabilities in various applications, including burn depth detection, dermatology, and vascular imaging^15^ by employing multi-spectral laser speckle contrast imaging (MS-LSCI). By doing so, we demonstrate that MS-LSCI enables high temporal resolution, real-time imaging, and the assessment of different penetration depths seen in Fig. 1.

### 1.1 Prior Works

#### 1.1.1 Multi-spectral non-invasive imaging and LSCI

Multi-spectral depth-resolved imaging has been previously proven effective through other imaging modalities such as spatial frequency domain imaging (SFDI)^16^^,^^17^ and diffuse optical tomography (DOT).^18^^,^^19^ SFDI utilizes patterned light to separate absorption and scattering properties in tissues, providing quantitative maps of optical properties over large areas. Similarly, DOT reconstructs 3D images of tissue absorption and scattering by measuring light transmitted through the tissue at multiple wavelengths, offering insights into tissue composition and function. However, these techniques face several limitations. SFDI and DOT systems typically require bulky and complex equipment, which hinders their miniaturization for portable or point-of-care applications.^16^ Moreover, achieving high specificity and sensitivity in standardized measurements using traditional imaging techniques can be challenging. These methods often have limited penetration depths, especially in highly scattering tissues, restricting their effectiveness for deep tissue imaging. Previous studies have also explored the use of multiple lasers in LSCI for various applications. Ziegler et al.^20^ utilized multi-wavelength LSCI to evaluate skin properties, demonstrating its effectiveness in skin diagnostics. Similarly, Davis et al.^21^ employed Monte Carlo simulations to analyze scattering effects in LSCI, with a focus on how these factors influence flow dynamics within vascular tissue layers. Other works include the investigation of melanoma diagnostics as shown by Sdobnov et al.,^22^ but this work did not specify the penetration depths for each laser, making it difficult to determine the exact depth of penetration. Zhang et al.^23^ demonstrated dual-wavelength LSCI (dwLSCI) for stroke rehabilitation using 850 and 805 nm lasers; however, the small wavelength difference limited the ability to accurately decouple absorption from scattering effects. Dunn^4^ used six laser diodes spanning 560 to 610 nm for cerebral applications to calculate hemoglobin oxygenation and concentration changes, but this approach is primarily limited to cerebral applications.

#### 1.1.2 Depth-resolved LSCI

Recent works on LSCI have built on these approaches and tried various approaches to resolve depth through mathematical models and deep learning techniques. A method based on depth-dependent contrast approaches by Chen et al.^24^^,^^25^ has been used for blood flow reconstruction and depth assessment. Maity et al.^26^ use a line scanning source-detector to investigate speckle contrast as a function of source-detector separation to construct a 3D blood flow map using convolution-based inverse algorithms. However, these methods suffer from parameter re-estimation for their Monte Carlo simulations, scanning time limitations, and complex hardware for hand-held use due to movements and motion artifacts.

In this work, we demonstrate the use of multi-spectral imaging in LSCI for depth-resolved imaging through the utilization of both visible and near-infrared lasers without any need for mechanically moving/scanning parts. Although previous studies have explored multi-spectral LSCI imaging for enhanced capture capabilities,^27^ our approach investigates depth-resolved imaging by combining multi-spectral data acquisition and wavelength-specific depth profiling.

## 2 Theory and Principles: Laser Speckle Contrast Imaging (LSCI)

### 2.1 Basic Principles of LSCI

When coherent light, such as that emitted by a laser, interacts with a rough or scattering surface, it produces a distinctive speckle pattern of randomly distributed bright and dark spots.^3^ This occurs due to the scattered light waves undergoing constructive and destructive interference, leading to fluctuations in light intensity at various points on the surface.

The size and contrast of the speckles in the pattern depend on several factors, including but not limited to the distance between the surface of the material and the lens capturing the scattered light, the wavelength of the light, and the scattering properties of the material.^15^ The speckle pattern itself is sensitive to changes or motion occurring in the illuminated material. When there are changes to optical properties, such as blood flow, the speckle pattern changes, and shifts. The variations in the speckle pattern can be captured and analyzed to extract information about dynamic movements, such as blood flow, occurring in the laser-illuminated area.

The speckle phenomenon can be mathematically represented by the equation

$$K=\frac{\sigma }{\left\langle I\right\rangle },$$ (1)

where K represents the local speckle contrast and is defined as a ratio of the standard deviation, σ, to the mean speckle pixel intensity, I. This speckle contrast can be obtained using various methods, including spatial and temporal contrast algorithms.^28^

As previously mentioned, generating reliable LSCI images requires the careful tuning of several parameters that have an influence on the laser speckle patterns. Among these are the laser power, the exposure time of the camera, the frame rate, the speckle size, and the image processing techniques.^15^

The speckle contrast obtained in LSCI is also dependent on the absorption coefficient ($\mu_{a}$) and reduced scattering coefficients ($\mu_{s}^{\prime }$).^29^ These parameters depend on the wavelength (λ) of light used for imaging.

### 2.2 Visibility Ratio

In this study, a critical parameter is the visibility ratio, $V_{r}$,^25^ which measures contrast within the speckle pattern. It is defined as

$$V_{r}=\frac{\left\langle I_{\max}\rangle -\langle I_{\min}\right\rangle }{\langle I\rangle_{st}}.$$ (2)

To compute $V_{r}$ accurately, we employed a sliding window approach. This sliding window, illustrated in Fig. 1, is a 7×7 spatial analysis window that moves across the speckle images. At each step, the maximum and minimum intensities, $I_{\max}$ and $I_{\min}$, are extracted within the window. These local intensity variations provide critical information for evaluating spatial contrast. Although $V_{r}$ serves as a measure of visibility and is used in this work as a depth-resolved metric, it can be considered analogous to the calculated speckle temporal contrast since both are derived from intensity variations. This aligns with the principles underlying calculated speckle temporal contrast, which also relies on the variation of intensities over time to compute flow or structural features.^30^

## 3 Methods

### 3.1 Monte Carlo Simulations

Monte Carlo simulations were employed to assess the light propagation through the tissue phantoms used in this study. The simulations were conducted using the Monte Carlo Command Line (MCCL),^31^ which calculates the fluence distribution within the tissue. The tissue phantom was modeled in MCCL using optical properties derived from the work of Ayers et al.^32^ The optical properties used in our experiment can be seen in Table 1.

**Table 1:** Optical properties for Monte Carlo simulations derived from Ayers et al.^32^

Wavelength (nm)	Absorption coefficient $\mu_{a}$ (1/mm)	Reduced scattering coefficient $\mu_{s}^{\prime }$ (1/mm)
450	0.015	1.8
515	0.014	1.7
658	0.012	1.1
850	0.010	0.9

Although the software does not support direct speckle contrast simulations, fluence plots were utilized to understand the distribution of light at different wavelengths within the tissue. These fluence plots provide crucial insights into light penetration, which is closely related to the speckle contrast observed in experiments.

**Fig. 2:**
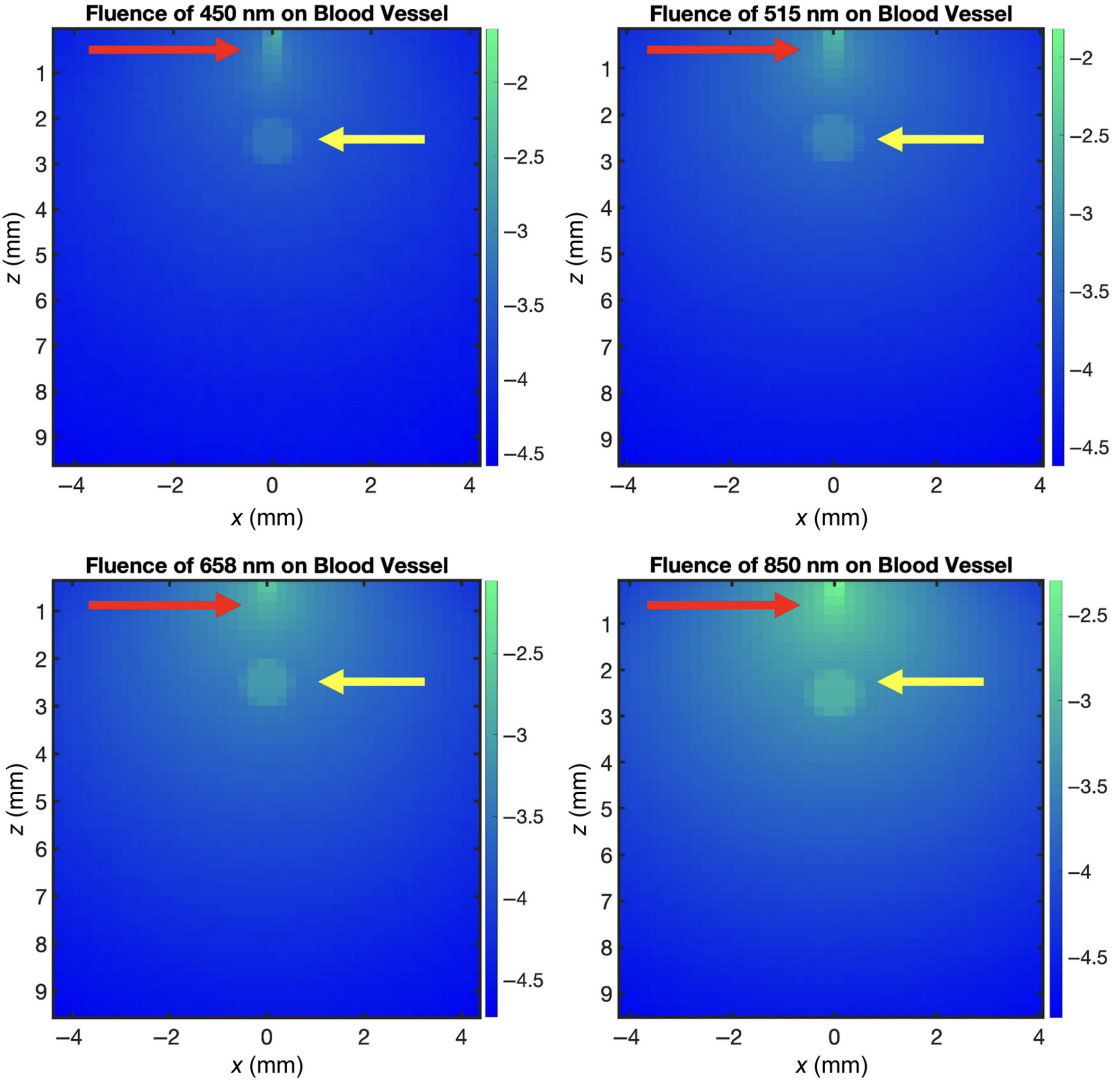
Simulated fluence comparison at various wavelengths on a blood vessel (450, 515, 658, and 850 nm), showing increasing depth of tissue penetration (the color bar is in $\log_{10}$ scale). Red arrows indicate the light source; yellow arrows point to the blood vessel’s location.

A 1,000,000 photon point source was defined on the surface of the phantom to simulate the interaction of laser light with the tissue. The simulations were run at each of the four wavelengths tested: 450, 515, 658, and 850 nm. These wavelengths were chosen to cover a broad spectrum from visible to near-infrared light, each having different penetration capabilities and interactions with tissue components. The results of these simulations are illustrated in Fig. 2. The red arrows in the figure indicate the light source, whereas the yellow arrows point to the location of the blood vessel. The fluence maps for each wavelength show how light penetrates and spreads through the tissue, with longer wavelengths generally penetrating deeper. The simulation results were validated against experimental data, demonstrating that regions with higher fluence correlate well with areas of increased speckle contrast in the LSCI images.

The results of these simulations are illustrated in Fig. 2. The red arrows in the figure indicate the light source, whereas the yellow arrows point to the location of the blood vessel. The fluence maps for each wavelength show how light penetrates and spreads through the tissue, with longer wavelengths generally penetrating deeper.

### 3.2 Tissue Phantom Fabrication

**Fig. 3:**
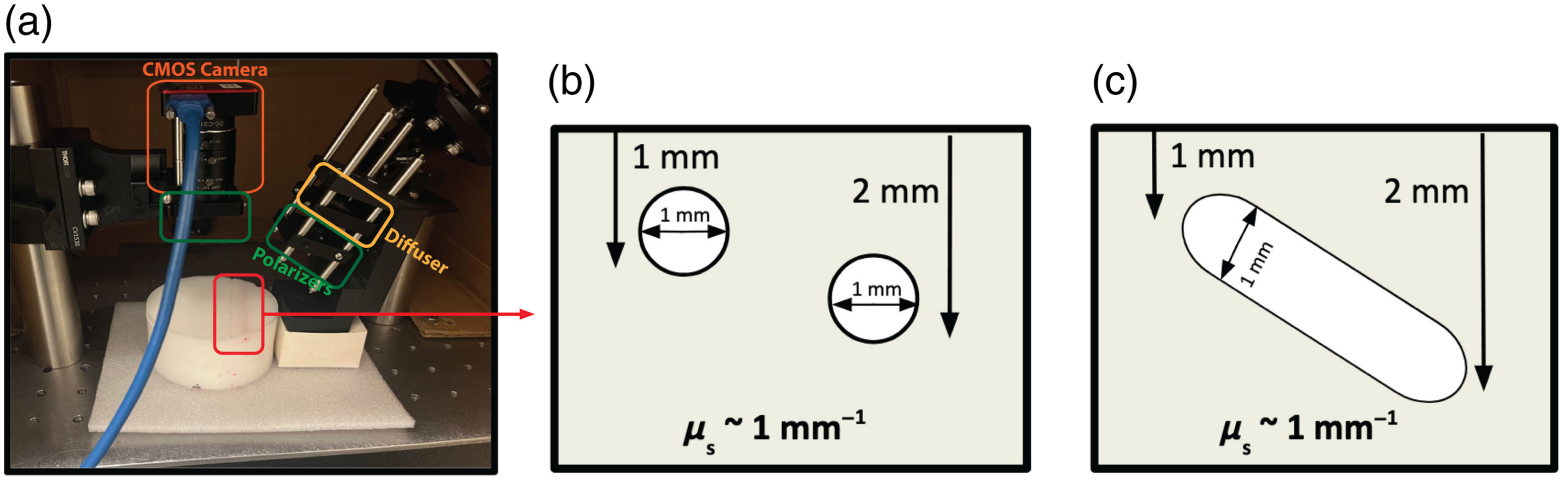
Experimental setup (a) with horizontal tissue phantom cross section (b) and calibration tissue phantom cross section (c).

To simulate different penetration depths and enable controlled experiments for assessing the capabilities of MS-LSCI, a polydimethylsiloxane (PDMS) tissue phantom was fabricated based on the work of Ayers et al.^32^ [Fig. 3(a)]. This horizontal phantom was fabricated such that two 1-mm-wide openings are built into the mold itself. These are spaced 1 cm apart, with the more superficial layer being embedded 1 mm deep, and the second layer placed 2 mm deep. These are spaced to mimic microvasculature structures and different skin layers in human tissue [Fig. 3(b)]. The calibration phantom used in this study was optimized for near-infrared wavelengths to simulate tissue optical properties. However, it is important to acknowledge that the phantom’s properties do not extend fully into the visible spectrum, where absorption can be higher.^33^ Consequently, the penetration depth estimated in the visible wavelength range may not perfectly replicate what would be expected in living tissue. This limitation should be considered when interpreting results derived from visible wavelengths, as the phantom cannot fully capture the optical complexities of real biological tissue at these shorter wavelengths.

To further evaluate the depth assessment capabilities, a second tissue phantom, referred to as the calibration phantom was fabricated. This phantom featured a sloped channel embedded at varying depths within the phantom material, designed to simulate different penetration depths along its length. This setup allowed for the assessment of how well the MS-LSCI technique could resolve flow at different depths along a continuous slope. The calibration phantom is depicted in the updated Fig. 3(c) for better visualization. The absorption coefficient ($\mu_{a}$) and reduced scattering coefficient ($\mu_{s}^{\prime }$) for these wavelengths were referenced and extrapolated based on the work of Ayers et al.^32^ These values are representative of typical biological tissues and were chosen to accurately simulate the conditions within the tissue phantom used for MS-LSCI. In this study, we use a sliding window of 7×7  pixels to calculate the maximum and minimum intensities across spatial regions. The visibility ratio was calculated as defined previously, and the inverse square $1/Vr^{2}$ was analyzed to detect the presence and estimate the relative depth of structures within the calibration phantom, as shown in the horizontal tissue phantom cross-section in Fig. 3(b). Peaks in $1/Vr^{2}$ were evaluated to determine if they consistently corresponded to the known depths of the calibration tube and the horizontal phantom tubes. If the peaks match the tube’s known depth profile, then $1/Vr^{2}$ can be used as a relative measure of depth for the two-layer horizontal phantom experiment.

### 3.3 Experimental Setup Used for MS-LSCI

**Fig. 4:**
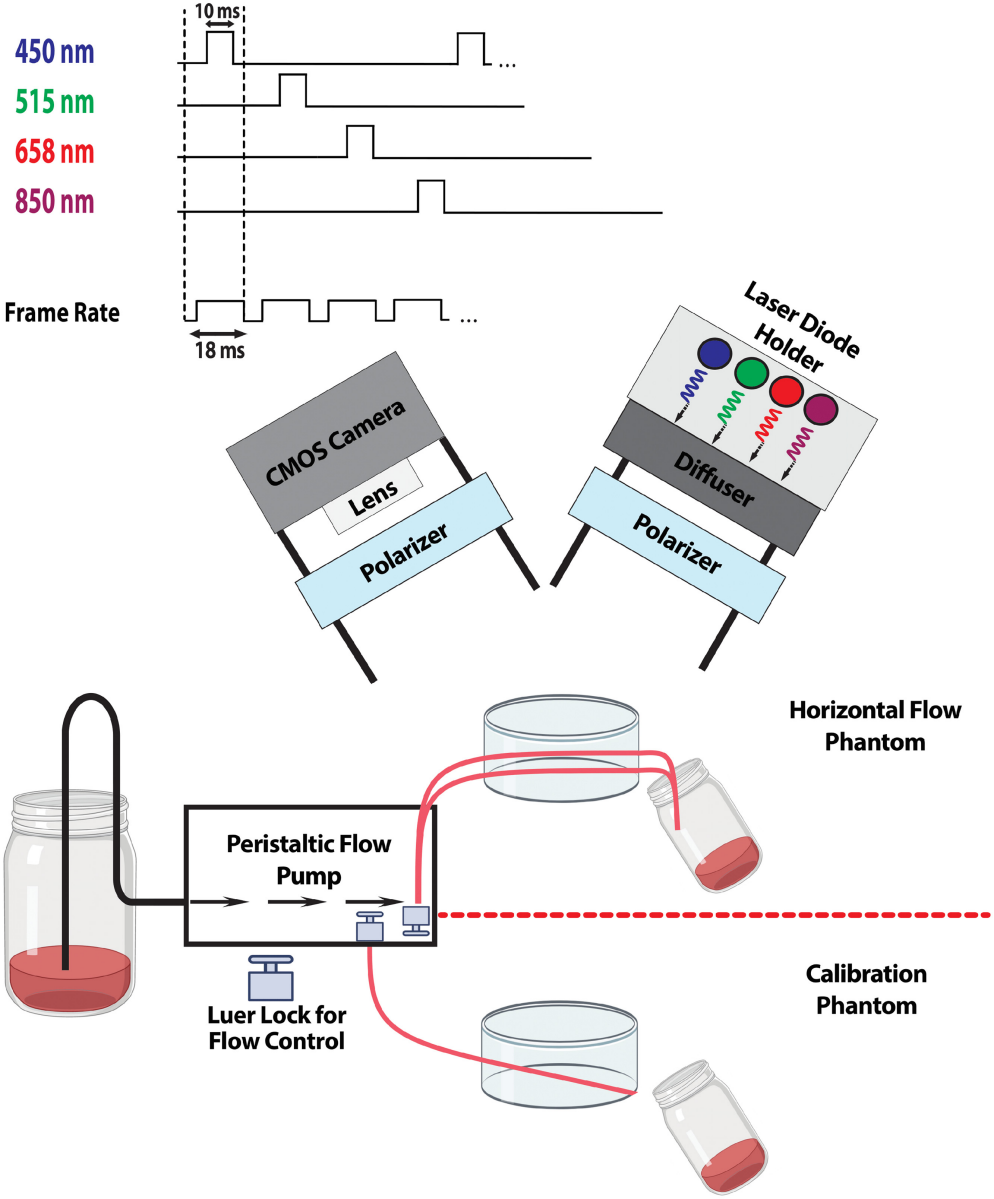
Experimental setup diagram including illumination and imaging path components, as well as timing of image capturing and laser pulsing.

The experimental setup for the MS-LSCI device includes laser sources with specific wavelengths to generate light-phantom interactions and speckle patterns (Fig. 4). We use four TO-can laser diodes from ThorLabs Inc.: 450 nm (PL450B), 515 nm (L515A1), 658 nm (HL6501MG), and 850 nm (L850P25).

The optical power density at the surface of the tissue phantom is $0.5\;\mathrm{mW}/{\mathrm{cm}}^{2}$ for each laser diode. To distribute the light uniformly across the surface of the tissue phantom, a 50-deg circle pattern engineered diffuser (Thorlabs ED1-C50) is placed near the laser sources, as shown in Fig. 4. The diffuser polarizes the light randomly, and as a result, a linear polarizer (LPVISE100-A, LPNIRE100-B) is placed immediately after to reduce unwanted reflections resulting from the randomly polarized light by the diffuser.

To capture the speckle patterns, the imaging system utilizes a CMOS camera (Thorlabs CS165MU1). The camera has a maximum resolution of 1440×1080 with a frame rate of ∼35 frames per second (fps) at this resolution. The pixel dimensions of the CMOS camera are 3.45  μm by 3.45  μm. An adjustable 4 to 12 mm focal length C Mount lens (INPERIUS) is also used, with a maximum aperture size of f/1.6. For this experiment, the camera was set to a frame rate of 55 frames per second with an image resolution of 700×700  pixels. To focus on the flow dynamics of the tissue phantom and reduce unwanted reflections, a cross-polarized setup was used. A C-mount lens was placed directly below the Thorlabs camera, with a polarizer placed near the CMOS sensor.

The minimum resolvable speckle size can be related through the equation

$$d_{\min}=1.22(1+M)(f/\#)\lambda ,$$ (3)

where $d_{\min}$ is the minimum speckle size, M is the magnification, f/# is the f-number, and λ is the wavelength.^34^ The speckle size $d_{\min}$ should be at least twice as large as the pixel size. In all cases for all four lasers, the pixel size of the camera is sufficiently large to accurately capture the speckle, as seen in Table 2.

**Table 2:** Calculated speckle sizes for different wavelengths (in microns).

Using Magnification M=8 and f-number f/#=1.6	
Wavelength λ (nm)	Speckle size $d_{\min}$ (μm)
450	7.920
515	9.065
658	11.582
850	15.012

We positioned the laser sources at a 30-deg angle to the normal to direct the emitted laser light onto the tissue phantom. The tissue phantom, which was fabricated to simulate different penetration depths as mentioned earlier, was placed ∼10  cm below the lens. This provided a field of view suitable enough to illuminate the entire phantom uniformly of ∼4  cm×4  cm. Simulated blood (VATA 2494) was passed through a peristaltic flow pump (Kamoer DIPump550) at a flow rate of 23  mm/s into the channels of the tissue phantoms.^26^^,^^35^

During image acquisition, the camera was set to record the speckle patterns with a fixed exposure time of 10 ms. Two-hundred raw 16-bit TIF images were captured for each wavelength and were used for temporal contrasting of the raw images. A Matlab script^36^^,^^37^ is employed for the temporal contrast algorithm, taking the raw captured TIF images as inputs.

## 4 Results

The results, detailed below, show how light penetration and speckle contrast depend on depth using MS-LSCI. Monte Carlo simulations first estimate how deep the light can penetrate for each wavelength in the tissue model. Next, we analyze the experimentally captured speckle data through raw images processed over time. The 450 and 515 nm wavelengths showed less penetration depth, clearly showing flow at 1 mm but not at 2 mm. By contrast, the 658 and 850 nm lasers showed penetration and visibility at both 1 and 2 mm depths.

Further analysis used a calibration model and a horizontal two-layer model to validate the depth assessment capabilities of the MS-LSCI technique. The calibration model, with varying depths, established a baseline for how $1/Vr^{2}$ peaks correspond to known depth changes. This baseline was used to analyze the horizontal two-layer model, which had layers at 1 and 2 mm depths. To further validate the depth assessment, a more complex tissue model was created with layers at 1 and 2 mm depths and flow crossing diagonally. The inverse square of the visibility ratio from the horizontal flow model was compared with the visibility ratio from the calibration model, allowing the separation of shallow and deep structures within the tissue model. This comparison effectively distinguished depth-specific flow characteristics, improving the accuracy and reliability of the depth assessment.

A wavelength mixing technique is implemented where we combine raw data from multiple wavelengths to leverage their distinct scattering and penetration properties. We calculate a difference map between pairs of wavelengths, emphasizing changes by contrast at different depths. This method ensures that structures appearing faint or indistinguishable at a single wavelength become more visible when the mixed-wavelength data is analyzed together. This technique was applied to both calibration phantoms and experimental data to validate its effectiveness.

**Fig. 5:**
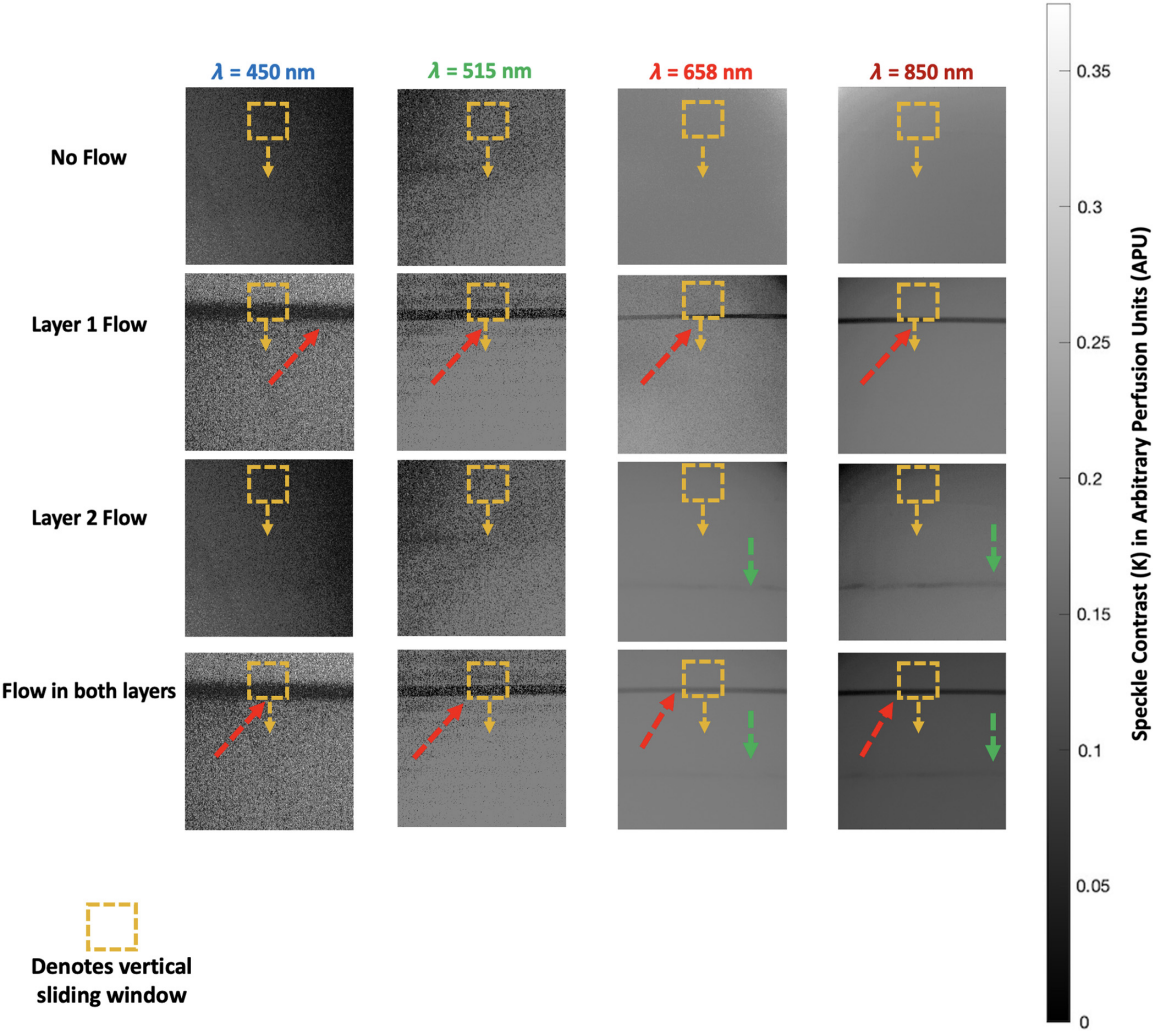
Speckle contrast images captured at 450, 515, 658, and 850 nm wavelengths under different flow conditions. Red arrows indicate 1 mm layer flow, while green arrows indicate 2 mm layer flow.

The multi-wavelength data for each flow state was done by calculating the speckle temporal contrast to generate LSCI flow maps, with visibility for the 1-mm layer flow being seen from all four wavelengths, and 2 mm layer being seen from the 658 and 850 nm lasers. The non-homogeneity observed in the speckle contrast maps across different wavelengths in Fig. 5 is attributed to the combination of varying optical properties of the wavelengths and the optical setup. Shorter wavelengths, such as 450 nm, exhibit higher scattering and are more sensitive to surface features, leading to greater variability in speckle patterns. By contrast, longer wavelengths, such as 850 nm, penetrate deeper into the medium, resulting in smoother contrast maps. Although camera settings were adjusted to optimize the laser intensity at the surface and maintain consistent pixel intensity levels across all wavelengths, intensity gradients were present in the raw images, likely caused by uneven illumination from the four laser diodes.

### 4.1 $1/Vr^{2}$ Results for 1 and 2 mm Flow Cases

**Fig. 6:**
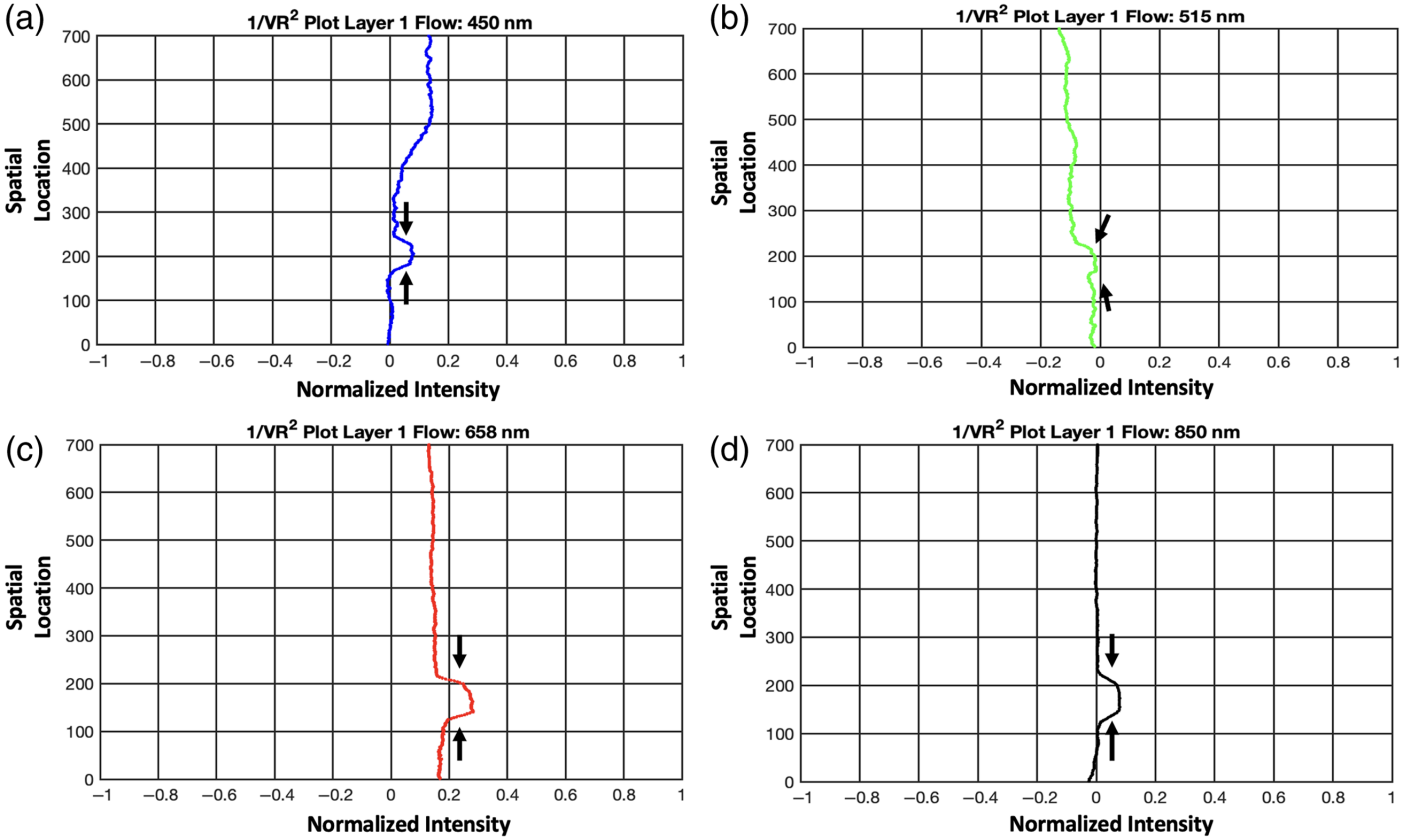
The $1/Vr^{2}$ plots with blood flow at 1 mm depth for 450 nm (a), 515 nm (b), 658 nm (c), and 850 nm (d).

The speckle contrast images obtained from MS-LSCI reveal clear visibility of the flow at both the 1 and 2 mm depths. The $1/Vr^{2}$ plots in Figs. 6(a)–6(d) display distinct peaks that align with the flow positions, particularly for the 1 mm depth. These peaks represent critical indicators of MS-LSCI’s sensitivity to changes in depth. Specifically, the $1/Vr^{2}$ peaks highlight the system’s ability to detect flow at subsurface levels, confirming the technique’s effectiveness at identifying features at varying depths. For the 1-mm depth, these peaks are consistently visible across all wavelengths used in the analysis, as shown in Figs. 6(a)–6(d).

**Fig. 7:**
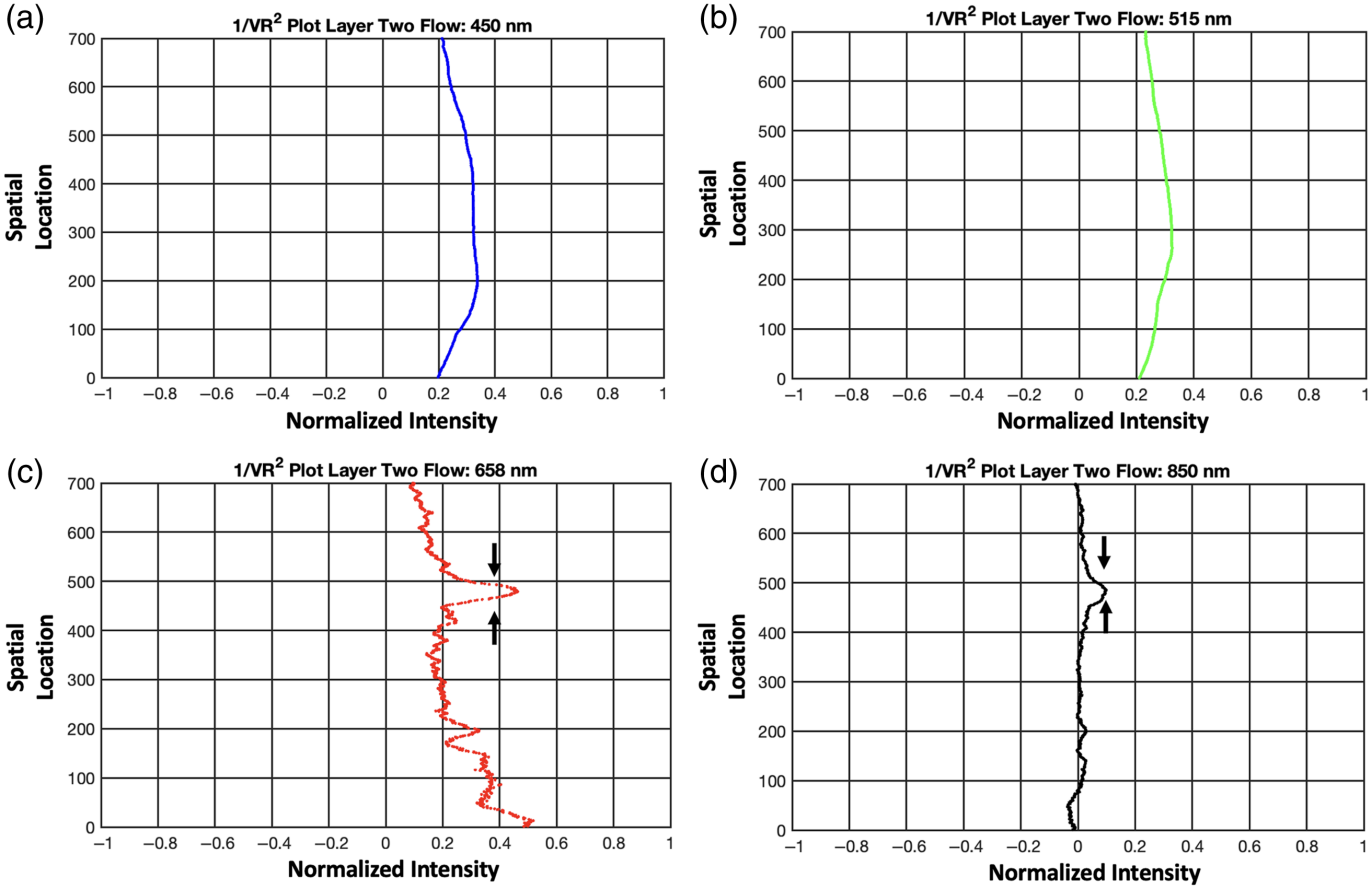
The $1/Vr^{2}$ plots with blood flow at 2 mm depth for 450 nm (a), 515 nm (b), 658 nm (c), and 850 nm (d).

However, at the 2-mm depth, the results diverge across the spectrum: while the 658 nm and 850 nm wavelengths continue to show visibility of the flow, the 450 and 515 nm wavelengths do not exhibit the same level of discernibility, as shown in Figs. 7(a)–7(d). To address these inherent limitations, we introduce a strategic contrast enhancement technique that combines information from multiple wavelengths, an approach referred to as wavelength mixing. This technique enhances the estimation accuracy of subsurface structures, even at wavelengths where penetration or contrast is typically poor. Wavelength mixing is discussed further in the next section, where its role in improving depth sensitivity and contrast is elaborated.

### 4.2 Correlation Plots for $1/Vr^{2}$ Between Calibration Phantom and Two Depth Horizontal Phantom

**Fig. 8:**
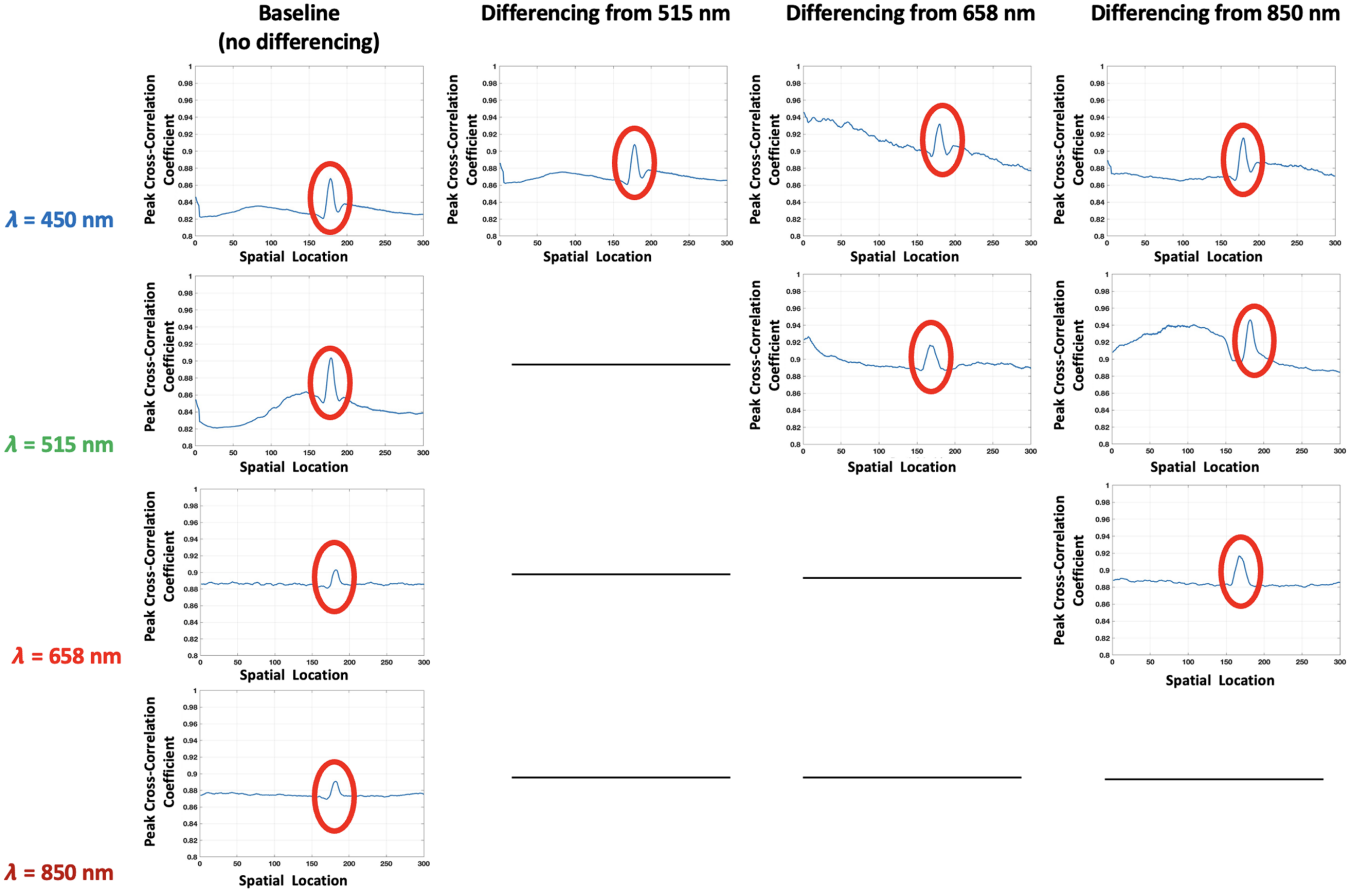
Depth-resolved cross-correlation for the 1 mm depth flow case, demonstrating the sensitivity of multi-wavelength laser speckle contrast imaging to changes in penetration depth using different laser wavelengths.

**Fig. 9:**
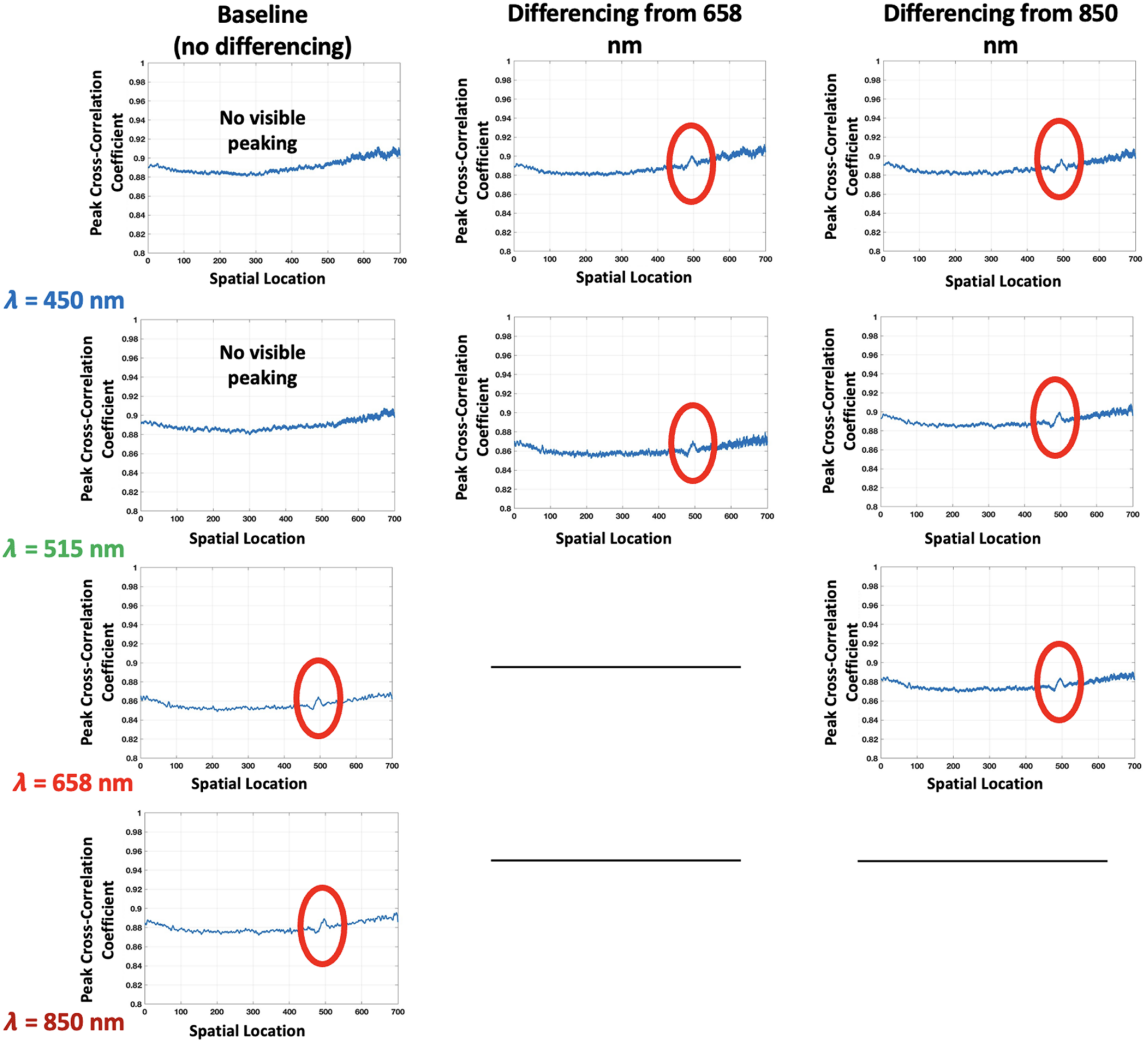
Depth-resolved cross-correlation for the 2 mm depth flow case, demonstrating the sensitivity of multi-wavelength laser speckle contrast imaging to changes in penetration depth using different laser wavelengths.

Subsequent analyses focused on correlational studies between the calibration phantom and a two-depth horizontal phantom to validate MS-LSCI’s capability for accurate depth assessment. The calibration phantom, with its gradient of known depths, established a reference for understanding the behavior of $1/Vr^{2}$ peaks in relation to depth variations. This benchmark was crucial in assessing the horizontal phantom, which was structured with distinct layers at 1 and 2 mm. The correlation plot for the layer one flow case, seen in Fig. 8, distinctly shows that at 1 mm, all wavelengths corresponded well with the $1/Vr^{2}$ profile, indicating a strong correlation with the known depth with peaking circled in red. However, at 2 mm, the correlation was more pronounced with the 658 and 850 nm lasers as seen in Fig. 9.

In Tables 3 and 4, we analyze the effectiveness of different wavelength combinations for assessing flow at depths of 1 and 2 mm, respectively. The reduced full-width half maximum (FWHM) values, especially in combinations such as 450 nm with 515 nm for shallow (1 mm) and 658 nm with 515 nm for deeper (2 mm) regions, demonstrate the capacity of these spectral mixes to enhance depth resolution. The results from Table 3 indicate that the blue and green wavelengths enhance the imaging at shallow depths. Conversely, Table 4 shows that red and green, as well as red and NIR, are effective at deeper depths, likely due to their improved penetration capabilities. By employing wavelength mixing through the contrast enhancement technique, the clarity of the correlation plots improves.

**Table 3:** Full width half maximum (FWHM) calculations for 1 mm correlation.

Wavelength (nm)	Base (no diff.)	Diff. from 515 nm	Diff. from 658 nm	Diff. from 850 nm
450	20 px	**14 px**	25 px	24 px
515	19 px	N/A	20 px	**16 px**
658	20 px	N/A	N/A	**16 px**
850	21 px	N/A	N/A	N/A

*Note*: FWHM values are highlighted (bold) when they represent the highest resolution achieved in each wavelength setup (lowest FWHM correlating to sharpest peaks).

**Table 4:** Full width half maximum (FWHM) calculations for 2 mm correlation case.

Wavelength (nm)	Base (no diff.)	Diff. from 658 nm	Diff. from 850 nm
450	No Peak	**18 px**	20 px
515	No Peak	**11 px**	15 px
658	15 px	N/A	**12 px**
850	12 px	N/A	N/A

*Note*: FWHM values are highlighted (bold) when they represent the highest resolution achieved in each wavelength setup (lowest FWHM correlating to sharpest peaks). “No peak” indicates no detectable peak was observed under the given conditions.

### 4.3 MS-LSCI for Hand Imaging

The correlation techniques derived from Lewis and Franco^27^ involve selecting regions of interest (ROI) to assess the effects of wavelength combinations for sharper resolution and image enhancement. This method leveraged the differences in light absorption at specific wavelengths to enhance contrast and reveal deeper structures. Inspired by this approach, we applied similar principles to our study, using multi-wavelength analysis to improve depth resolution in our tissue phantom experiments. In our work, this is done by taking two raw TIF image stacks for processing. We first analyze the variations between these images, then calculate $1/Vr^{2}$, and finally apply temporal LSCI.

**Fig. 10:**
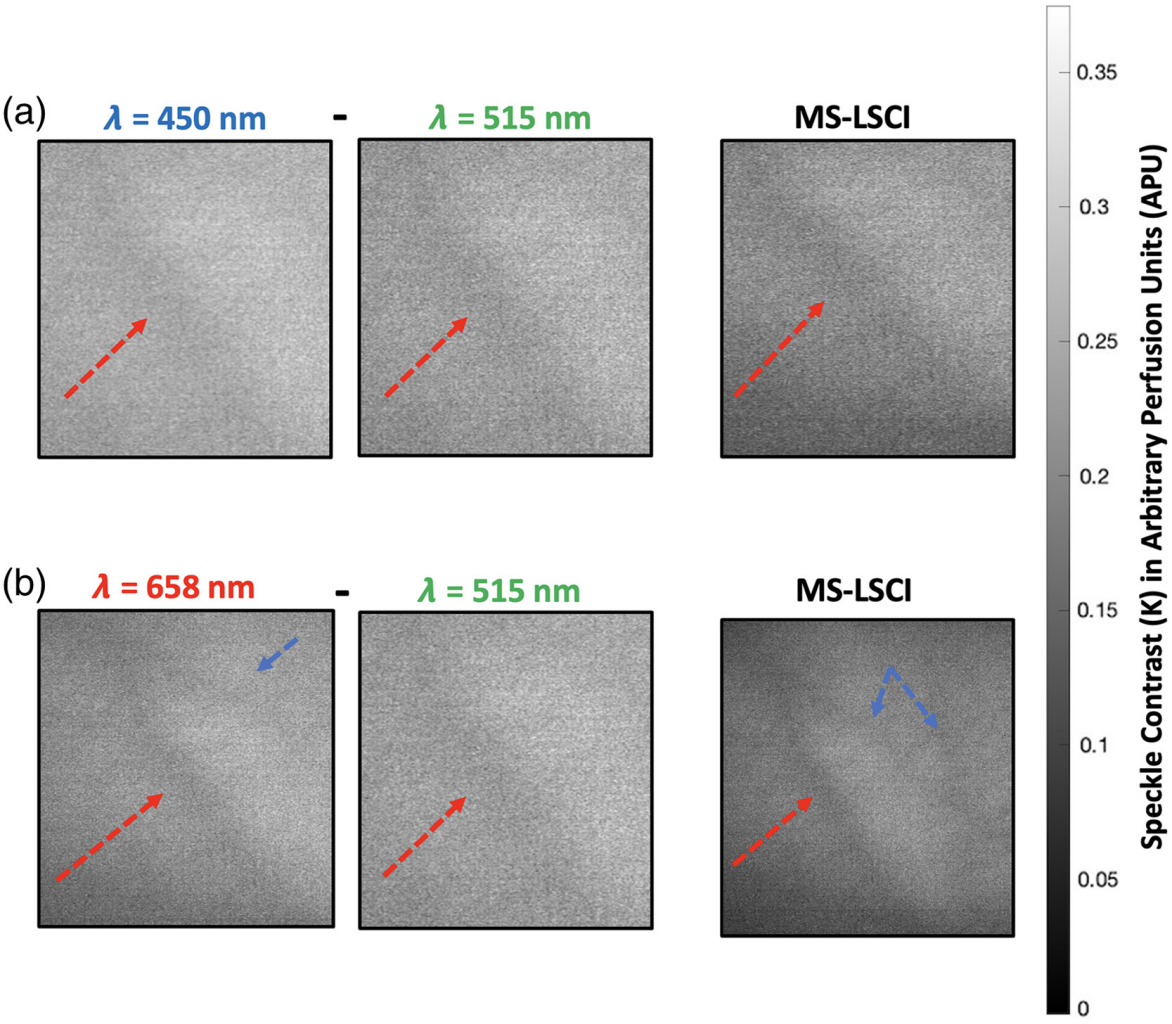
Enhanced LSCI image capture on human hand, where red arrow reveals structure seen by both blue and green wavelengths, and blue arrow reveals structures seen after wavelength mapping and mixing: panel (a) shows image combination for shallow flow assessment between blue and green. Panel (b) shows wavelength enhancing for deep flow assessment using red and green.

Figure 10 demonstrates the effectiveness of using MS-LSCI for assessing blood flow at different tissue depths on a human hand. The red arrow reveals structures seen by all wavelengths, whereas the blue arrow highlights structures seen after wavelength mapping and mixing. The combination of blue (515 nm) and green (450 nm) wavelengths enables assessing shallow flow, while for deeper flow assessment, wavelength enhancing is performed using red (658 nm) and green (515 nm) wavelengths. These results validate the effectiveness of multi-wavelength mixing in LSCI, enhancing depth resolution and contrast. Combinations of blue and green wavelengths optimally resolved features at shallower depths due to their complementary penetration capabilities, whereas red and green wavelengths were most effective for deeper regions, revealing structures not seen by the red wavelength alone.

## 5 Conclusion

In this study, we have demonstrated the application of MS-LSCI as a focused non-invasive technique for assessing penetration depth in tissue-mimicking phantoms, offering a new perspective on this approach. Monte Carlo simulations were initially performed to validate the expected penetration depths for the four laser wavelengths used: 450, 515, 658, and 850 nm. Experimental results from raw and calculated speckle temporal contrasted images confirmed that the visible wavelengths (450 and 515 nm) could resolve flow at a depth of 1 mm, whereas 658 and 850 nm wavelengths were capable of penetrating up to 2 mm depth in the tissue phantoms.

We introduced the use of the inverse square of the visibility ratio, $1/Vr^{2}$, as an effective metric for correlating speckle contrast with depth information. By analyzing $1/Vr^{2}$ profiles across a calibration phantom with known depth gradients, we established a baseline for interpreting the results from a two-layer horizontal phantom. The correlation plots revealed a strong agreement between $1/Vr^{2}$ peaks and the embedded flow positions at 1 mm depth for all wavelengths. At 2 mm depth, the correlation was more pronounced for the 658 and 850 nm lasers, whereas the visible wavelengths exhibited decreased signal quality due to their limited penetration capabilities.

To overcome this limitation, we proposed a technique of spectral mixing that improved the clarity of correlation plots and enabled more accurate depth localization, even for wavelengths with limited penetration. These results were further verified with LSCI images of a hand. This approach highlights the potential of MS-LSCI, in conjunction with advanced signal processing techniques, for non-invasive depth profiling in biomedical applications, such as burn wound imaging and vascular imaging.

Although we have used a simplistic approach of linear combination of multi-spectral raw images and a phantom calibration baseline, we plan to develop a more sophisticated approach using convolution-based inverse algorithms similar to Maity et al.^26^ in the future. The main challenges to doing so are the lack of existing datasets for ground truth validation and the lack of commercially available methods for measuring depth-resolved blood flow in the laboratory setting. By addressing these challenges, we aim to enhance the accuracy and reliability of MS-LSCI for clinical applications.

Although this study demonstrates the viability of multispectral LSCI for depth-sensitive imaging using a single set of phantom optical properties and one flow speed, we acknowledge that *in vivo* scenarios introduce greater complexity. In practical applications, the optical properties of tissues and flow speeds can vary significantly, posing challenges for consistent depth resolution and flow detection. Addressing these variations is critical for the advancement of this technique toward clinical or biological applications.

Future work will focus on expanding the scope of parameters, including varying flow speeds and optical properties, to better reflect *in vivo* conditions. Additional experiments with a wider range of phantoms and flow conditions will help to refine the technique and assess its robustness in more dynamic and heterogeneous environments. This will also include developing strategies to mitigate the challenges posed by differing tissue properties and flow dynamics. The goal is to evolve this proof-of-concept into a reliable and adaptable tool for real-world applications, ensuring that the method can handle the complexities of biological tissues.

Although the exact penetration depth values obtained from the phantom may not directly translate to *in vivo* conditions, the general patterns of contrast change provide meaningful insight into perfusion dynamics. As such, we believe that these limitations introduce only a minor source of error, without significantly affecting the conclusions drawn from the *in vivo* data. Future work will focus on refining the calibration phantom to capture both NIR and visible optical properties, enhancing its accuracy and applicability for real-world biological tissue assessments.

**Fig. 11:**
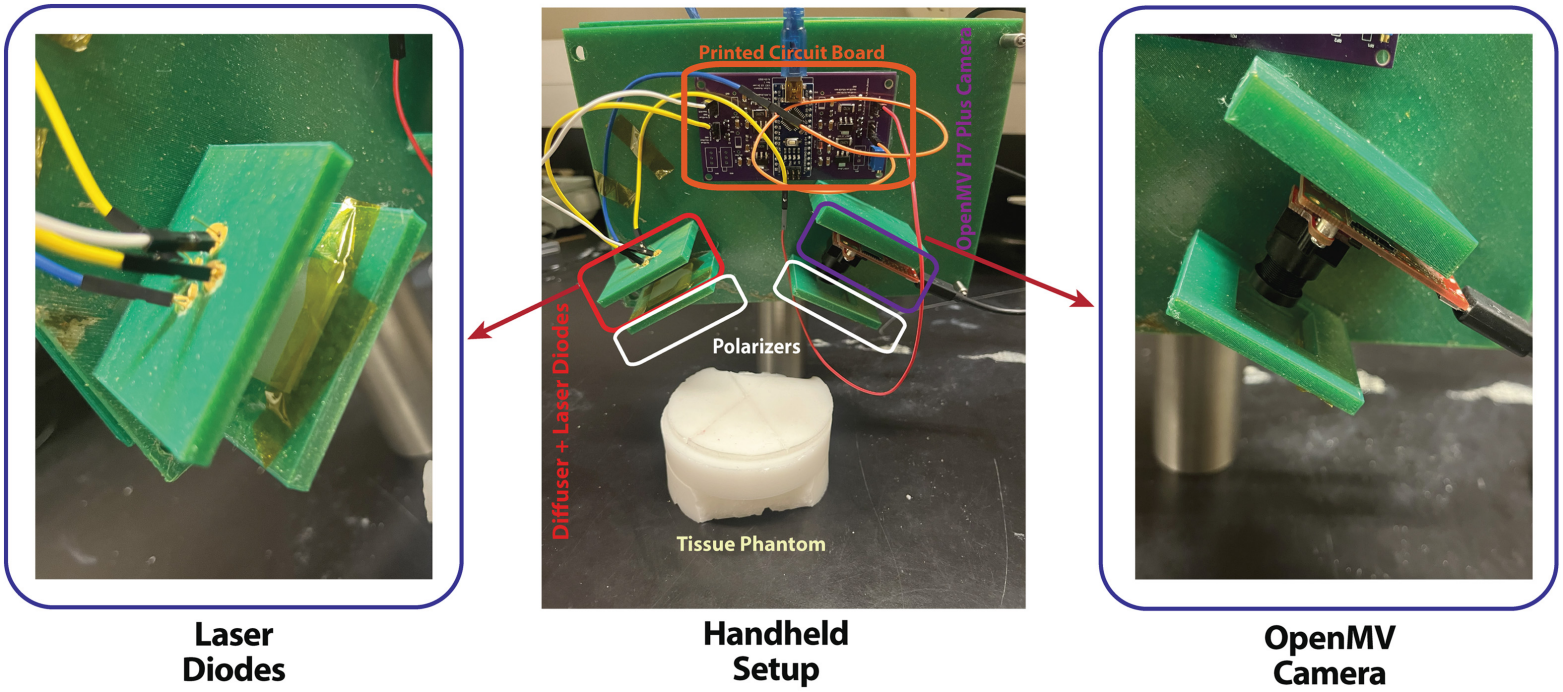
Handheld LSCI prototype device with close ups of laser diodes and OpenMV H7 Plus camera.

Other future work includes continuing the development of the miniaturized and handheld MS-LSCI device seen in Fig. 11, enabling *in vivo* experiments and clinical validation of the proposed techniques. The handheld device will facilitate further verification of the depth assessment capabilities demonstrated in this study, with computational algorithms being taken into account to remove the need for an inclined calibration tube, allowing for more realistic testing scenarios. In addition, efforts will be directed toward integrating the multi-wavelength laser sources and imaging components into a compact and user-friendly system. Successful integration and miniaturization will pave the way for conducting *in vivo* experiments and eventually exploring potential clinical applications of the MS-LSCI technology. These *in vivo* studies will provide valuable insights into the technique’s performance in real-world scenarios and aid in refining the methodology for optimal results.

## Data Availability

The code written during the current study is available from the corresponding author upon reasonable request.
